# Graph Theoretical Analysis of BOLD Functional Connectivity during Human Sleep without EEG Monitoring

**DOI:** 10.1371/journal.pone.0137297

**Published:** 2015-09-11

**Authors:** Jun Lv, Dongdong Liu, Jing Ma, Xiaoying Wang, Jue Zhang

**Affiliations:** 1 Academy of Advanced Interdisciplinary Studies, Peking University, Beijing, China; 2 School of Biomedical Engineering, Capital Medical University, Beijing, China; 3 Dept. of Pulmonary Medicine, Peking University First Hospital, Beijing, China; 4 Dept. of Radiology, Peking University First Hospital, Beijing, China; 5 College of Engineering, Peking University, Beijing, China; Institute of Psychology, Chinese Academy of Sciences, CHINA

## Abstract

**Background:**

Functional brain networks of human have been revealed to have small-world properties by both analyzing electroencephalogram (EEG) and functional magnetic resonance imaging (fMRI) time series.

**Methods & Results:**

In our study, by using graph theoretical analysis, we attempted to investigate the changes of paralimbic-limbic cortex between wake and sleep states. Ten healthy young people were recruited to our experiment. Data from 2 subjects were excluded for the reason that they had not fallen asleep during the experiment. For each subject, blood oxygen level dependency (BOLD) images were acquired to analyze brain network, and peripheral pulse signals were obtained continuously to identify if the subject was in sleep periods. Results of fMRI showed that brain networks exhibited stronger small-world characteristics during sleep state as compared to wake state, which was in consistent with previous studies using EEG synchronization. Moreover, we observed that compared with wake state, paralimbic-limbic cortex had less connectivity with neocortical system and centrencephalic structure in sleep.

**Conclusions:**

In conclusion, this is the first study, to our knowledge, has observed that small-world properties of brain functional networks altered when human sleeps without EEG synchronization. Moreover, we speculate that paralimbic-limbic cortex organization owns an efficient defense mechanism responsible for suppressing the external environment interference when humans sleep, which is consistent with the hypothesis that the paralimbic-limbic cortex may be functionally disconnected from brain regions which directly mediate their interactions with the external environment. Our findings also provide a reasonable explanation why stable sleep exhibits homeostasis which is far less susceptible to outside world.

## Introduction

Functional brain networks of human have been found exhibiting small-world topology by analyzing functional magnetic resonance imaging (fMRI) time series with graph theory [1]. The reasons why small-world topology is a promising model for large-scale brain networks are: (1) Our brain is a complex network on multiple spatial and time scales [2]; (2) Our brain owns the ability for both specialized or modular processing in local areas and distributed or integrated processing over the whole network [3,4]; (3) Our brain likely evolved to maximize efficiency at a minimal cost for effective information processing between different brain regions [5]. Small-world properties of our brain networks have helped to understand mechanism of cognitive function and even uncover clue to the pathology of neurodegenerative disorder such as schizophrenia [6], Alzheimer [7] and depressive disorder [8].

According to graph theory analysis of electroencephalogram (EEG) on particular frequency bands during sleep, Ferri found that both the neocortical connectivity and the small-world properties had increased [9,10]. fMRI studies on default mode network (DMN) have reported that there was a breakdown of the coupling between the anterior and posterior nodes during slow-wave sleep [11]. Moreover, Sämann found that the connection between hippocampus and the DMN had reduced at sleep onset [12]. In addition, incorporating fMRI with EEG, Spoormaker et al. [13] realized that thalamocortical connectivity was reduced at sleep onset and general connectivity was broken down in slow-wave sleep.

However, with EEG monitoring, subjects feel difficulty in getting to sleep, especially patients. Hence, in our study, we replaced EEG with peripheral pulse. Using the Talaraich atlas, brain regions are usually divided into neocortical system, paralimbic-limbic cortex and centrencephalic structure [14]. The neocortical regions include frontal areas 1–16,23,24; temporal areas 81–90; occipital areas 49–54; parietal areas 59–62 [14, 15]; opercular areas 17,18; the unimodal sensory such as fusiform gyrus 55,56; heteromodal sensory 63–66; and other areas such as the heschl gyrus 79,80 [16], the precuneus 67,68 and the cuneus 45,46 [17], the lingual gyrus 47,48 [18], precentral/postcentral/paracentral gyrus 1,2,57,58,69,70 [19] and calcarine fissure 43, 44 [20]. The centrencephalic cortex contain thalamus 77,78; caudate nucleus 71,72 and lenticular nucleus such as putamen 73,74 and pallidum 75,76 [14,15].

It is believed that paralimbic-limbic cortex usually encompasses brain regions that are involved in memory and the integration of autonomic functions, including subcortical structures such as the amygdala, hippocampus and basal forebrain, as well as cortical areas such as the parahippocampal and anterior cingulate cortices [21].

Nofzinger’s [22] study has reported that paralimbic cortices, including the anterior cingulate gyrus and parahippocampal gyrus, had a decreased activity by positron emission tomography (PET) monitoring during non-rapid eye movement (NREM) sleep relative to wakefulness, whereas other cortices did not. By non-invasive perfusion imaging strategy, Braun et al. [14] realized that this disengagement of paralimbic structures and the isolation of limbic structures (e.g. amygdala, hippocampus) from other cortices may promote the restorative function of NREM sleep. To exploit the changes of paralimbic-limbic structures with simpler method, here, by using graph theoretical analysis, we attempted to investigate the changes of paralimbic-limbic cortex between wake and sleep states, which could be reflected in small-world properties and functional connectivity of brain networks.

## Materials and Methods

### Participants

This study was approved by BioMed-X Research Center Ethical Review Committee of Academy for Advanced Interdisciplinary Studies, Peking University, Beijing, 100871, China. Participants have provided their written informed consents after the procedure had been fully explained.

Ten young and healthy subjects were recruited to sleep in MR scanner between 11pm-6am: five females and five males with a mean ± SD age of 23.8 ± 2.2 years. All subjects were right-handed and had no history of psychical disease and sleep disorder. In this study, the subjects have neither been deprived of sleep nor taken any sleeping pill. Data from 2 subjects were excluded for the reason that they could not fall asleep during the experiment.

### Image Acquisition

**Fig 1:**
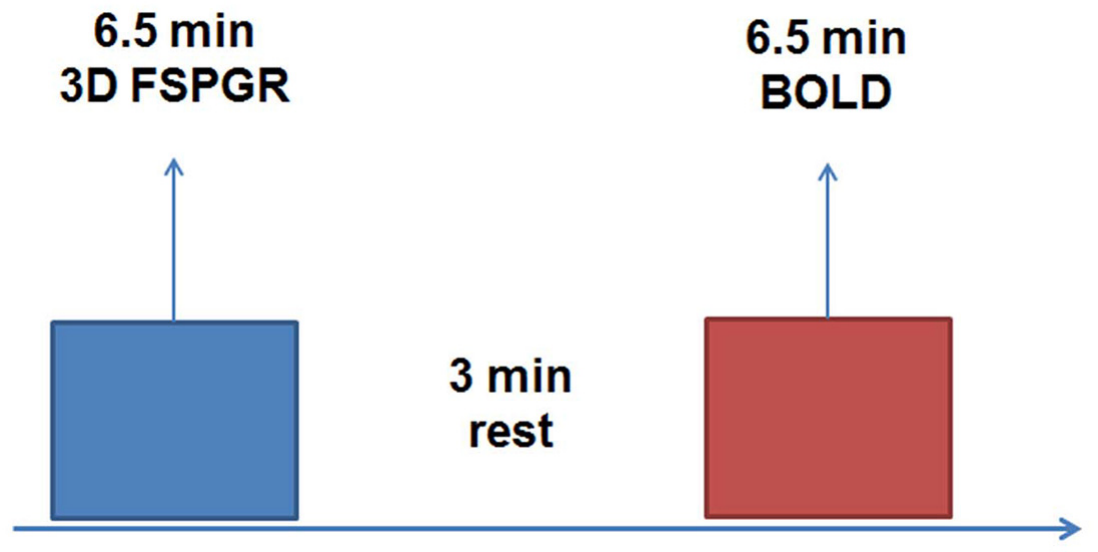
fMRI Scanning Process.

Every participant needed two MR scanning sessions which were performed at a 3T whole-body system (Signa Excite HD; GE Medical Systems, Milwaukee, WI) with an eight-channel head coil. The first scanning was carried out when subjects were awake. During the scanning process, subjects were asked to lie still with eyes open and not think initiatively. The scanning process is shown in Fig 1. T1-weighted structural images obtained through 3D FSPGR (fast spoiled gradient-echo dual-echo) (TR = 25 ms, TE = 4 ms, thickness = 2.0 mm with no gap, FOV = 230 mm^2^) were used for registration during the analysis of functional data. Blood oxygen level dependency (BOLD) based functional images were collected by using an echo-planar imaging (EPI) sequence: TR = 2,000 ms, TE = 30 ms, slices = 23, thickness = 3 mm, gap = 1 mm, FOV = 240 mm^2^, acquisition matrix = 64 × 64.

The second scanning was carried out to record subjects’ sleep state. Peripheral pulse signals were simultaneously obtained to identify the corresponding sleep periods of each subject. During our fMRI experiment, after the subject’s heart rate decreased and remained stable for a period of time, we sent the scanning bed into the scanner. In addition, we also patted the subject to ensure that he/she had fallen asleep. Then, we began to scan. The time each subject took before he/she went to sleep varied from person to person. It usually took about 1 hours. The peripheral pulse equipment we used is commercial equipment of the 3T MR whole-body system (Signa Excite HD; GE Medical Systems, Milwaukee, WI). The scanning process of sleep state is also shown in Fig 1. Each subject was scanned only once.

### Data Analyses

Data were processed by SPM8 (www.fil.ion.ucl.ac.uk/spm) and MATLAB. Power spectrum density (PSD) analysis technique of heart rate variability (HRV) extracted from peripheral pulse signals was used to ensure whether or not the BOLD data were in sleep state. BOLD images were first realigned to the first scan to correct for potential head movement between scans and then time corrected to compensate for delays associated with acquisition time differences. Finally, based on de-noise approach mentioned in many previous studies [1,23], functional images were spatially smoothed using a gaussian filter and temporally filtered (0.03–0.06Hz) [1, 24–27] to remove low-frequency drift and high-frequency physiological noises. The data sets preprocessed above were divided into 90 regions of interest (ROIs) (45 for each hemisphere) based on the AAL-atlas [28]. The mean time series of each region were then obtained by averaging the time series of all voxels in that area. The Pearson correlation coefficients between each possible pair of the regional residual time series were calculated, and a 90*90 correlation matrices were obtained for each subject. Then a Fisher’s r-to-z transformation was applied to the correlation matrices to improve the normality of the correlation coefficients, and the z-score matrices were obtained. Finally, each absolute z-score matrix was thresholded into an undirected binary graph network for further analysis using graph theoretical approaches with the nodes describing brain regions and the edges describing the links between the regions [29].

### Statistical analysis

In this study, the network degree was used for threshold measurement, which was from 21 to 35 to make the small-world attributes estimable and the resulting matrices sparse [23]. Meanwhile, 100 degree-matched random networks were generated [3,4,30,31] and several small-world parameters of the networks were obtained as well, including global efficiency (Eglob), local efficiency (Eloc), characteristic path length (L) and clustering coefficient (C) [29]. Meanwhile, we compared the small-world parameters (Cnet, Lnet, Eloc, Eglob) at each degree to evaluate the differences between the wake and sleep groups using a paired t-tests. A false discovery rate (FDR) of p<0.05 was used to correct the multiple comparisons.

### Visualization of functional brain networks

The Pajek software package (vlado.fmf.uni-lj.si/pub/networks/pajek) was used to to make binary connection matrices visualized. We made the brain regions which are in the same cortex together for ease of viewing.

## Results

**Fig 2:**
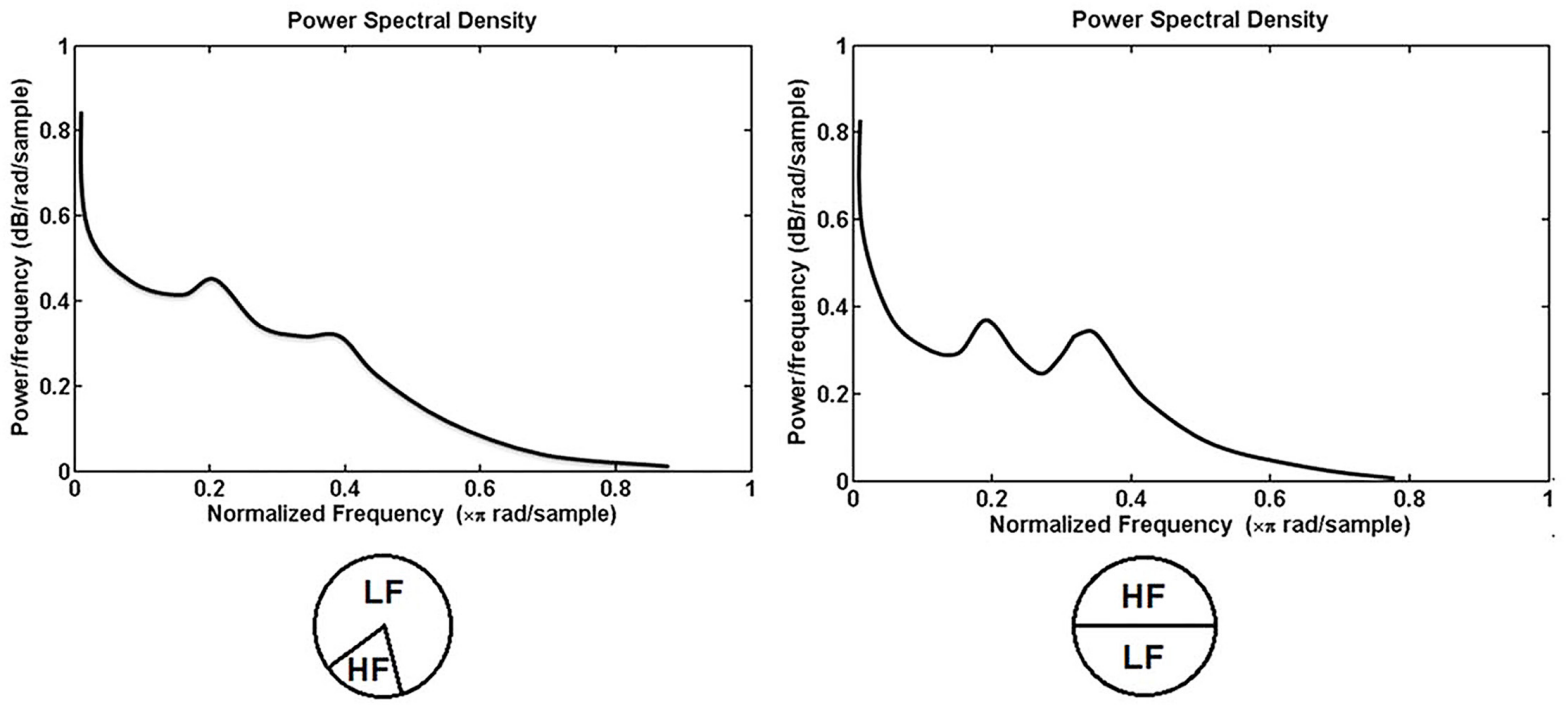
A typical result of a subject in wake and sleep state. (a) PSD of HRV is dominated by low-frequency components. (b) PSD of HRV is dominated by high-frequency components.

Fig 2 indicates the result of a typical subject which low-frequency (LF) / high-frequency (HF) ratio is different between wake and sleep state. When low frequency components play a dominant role in the power spectrum density (PSD) (Fig 2a), the subject is awake. Otherwise, if the value of LF/HF is close to one, it is illustrated that the subject is in sleep state (Fig 2b).

**Fig 3:**
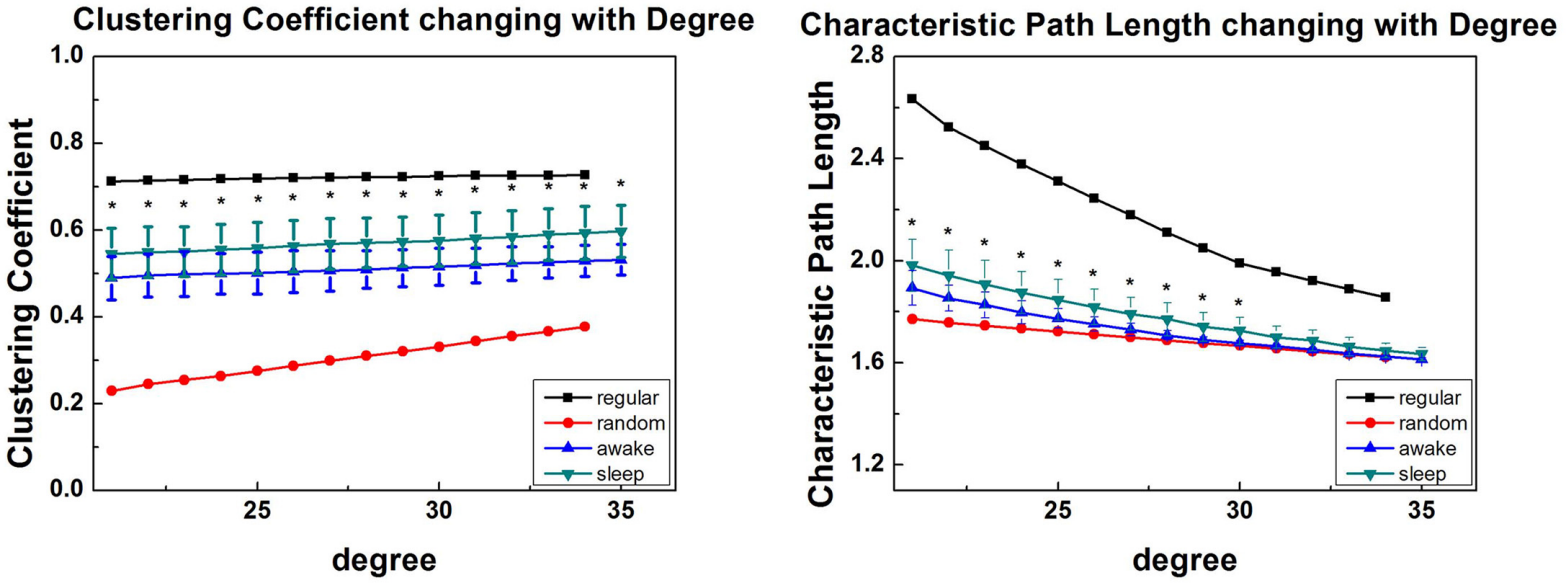
Group results of … (A) clustering coefficient, C_net_, and (B) characteristic path length, L_net_, for sleep group (green dots) and wake group (blue dots) as a function for degree. Error bars correspond to standard error of the mean. Black dots above indicate significant group difference (p<0.05, false discovery rate (FDR) corrected).

As shown in Fig 3, at the whole range of degree, the brain networks of the sleep group demonstrated notably higher clustering coefficient and significant longer characteristic path length compared with wake group, which reflected a trend toward a regular network. Statistical analysis revealed that there were significant differences in Cnet and Lnet between wake and sleep states (p<0.05, FDR corrected).

**Fig 4:**
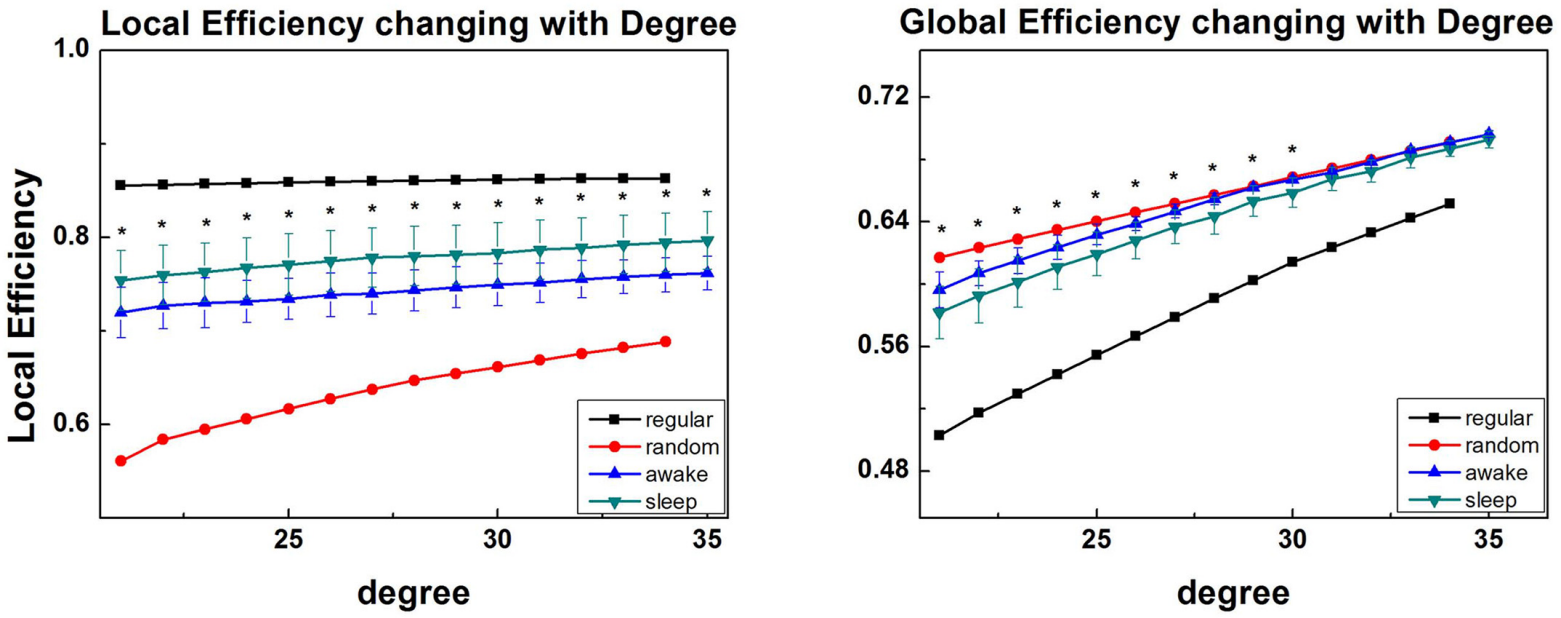
Group results of … (A) local efficiency, Eloc, and (B) global efficiency, Eglob, for sleep group (green dots) and wake group (blue dots) as a function of degree. Error bars correspond to standard error of the mean. Black dots above indicate significant group difference (p<0.05, FDR corrected).

It is found that, at the whole range of degree, the brain networks of the sleep group demonstrated remarkable higher local efficiency and significant lower global efficiency compared with wake groups (Fig 4), which also indicated a trend to the regular network. Throughout all the observations at each degree value, paired t-tests showed there exists a significant differences in Eloc and Eglob between wake and sleep states (p<0.05, FDR corrected).

**Fig 5:**
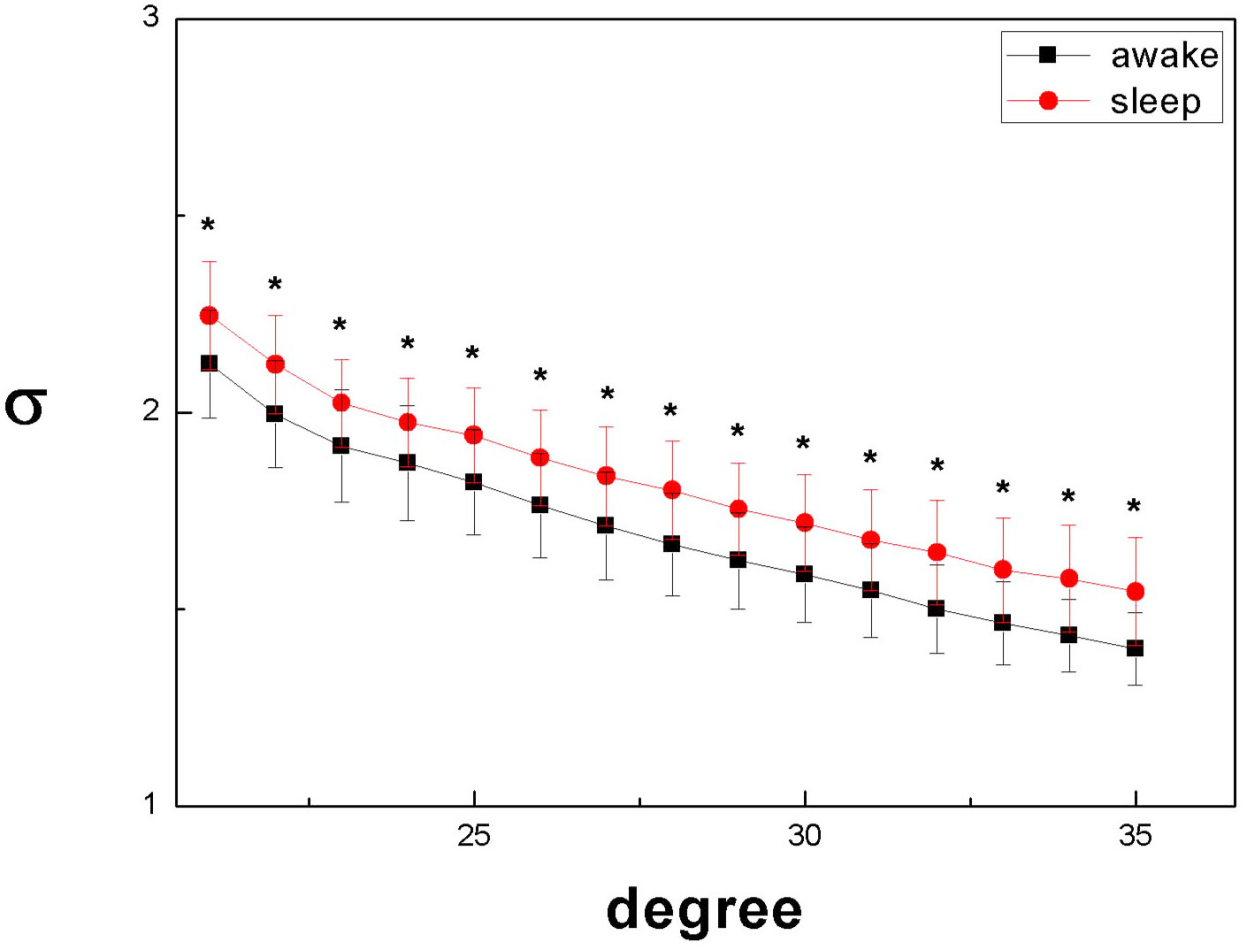
Small-world property σ during sleep states as compared to the wake state.

As shown in Fig 5, networks prove to exhibit higher small-world properties during sleep states as compared to the wake state.

**Fig 6:**
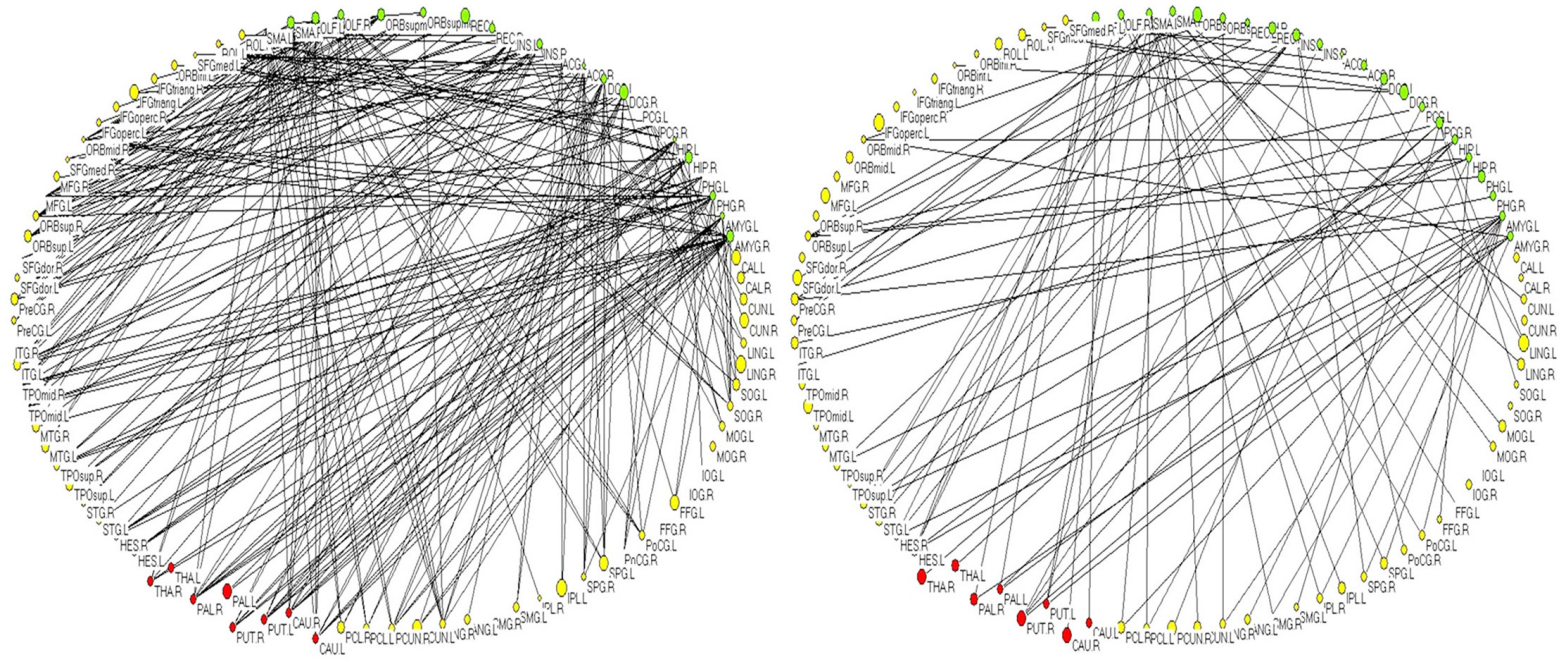
Connectivity graphs of wake and sleep state. The networks represent binary connections, locally organized by a layout algorithm implemented in the Pajek software package. The yellow nodes represent the neocortical system, the green nodes represent the paralimbic-limbic cortex and the red nodes represent the centrencephalic structure. (Left:wake, Right:sleep)

Differential connectivity graphs between wake state and sleep state are demonstrated in Fig 6. We realized that the paralimbic-limbic cortex of the right network had less links to other cortices compared with the left network.

**Fig 7:**
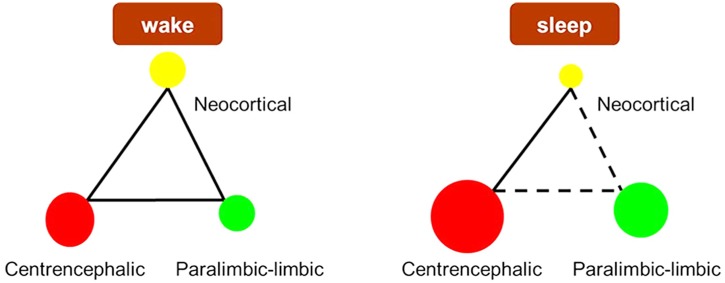
Schematic diagram of wake and sleep state. The yellow node represents the neocortical system, the green node represents the paralimbic-limbic cortex and the red node represents the centrencephalic structure. The size of the dot represents the value of the corresponding local efficiency. Dotted line represents connections reduced in number.

According to the figures acquired by Pajek software, the links in brain were simplified as Fig 7. We found that the local efficiency of paralimbic-limbic cortex and centrencephalic structure increased, whereas the value of neocortical system decreased.

## Discussion

People have realized that small-world properties of brain network have a direct relationship to many pathology of neurodegenerative disorder. However, we wonder whether there is a change in small-world properties of brain network when humans sleep. Our study investigated the changes of paralimbic-limbic cortex between wake and stable sleep states in the sight of small-world properties and functional connectivity of brain network.

In this study, to make the subject fell into natural sleep state more easily, we took three measures. First, subjects have neither been deprived of sleep nor taken any sleeping pill. Second, we did not send the scanning bed into the scanner until the subject went into sleep state. Third, subject did not wear uncomfortable EEG system. The scan of functional image was triggered by peripheral pulse signal.

Actually, Greene and Siegel’s [32] study has pointed out that people would have significantly lower heart rate in sleep state. Moreover, according to the study of Horovitz et al. [11], to facilitate stable sleep in the MRI environment; we performed this experiment between 2:00 and 6:00 am. Furthermore, Parker [33] demonstrated that in contrast to ECG gating, in which cardiac systole happens shortly after the R wave, peripheral pulse signal is a delayed representation of systole. More importantly, Maderwald et al. [34] finded that ECG signal was often affected by the challenges relative to the increased magnetohydrodynamic potential of flowing blood in high magnetic fields, which would cause inaccurate trigger signal. In that case, peripheral pulse was used. Therefore, in our study, it is rational to replace ECG gating with peripheral pulse.

Malliani [35] indicated that though parasympathetic mechanisms probably contribute to the power involved in the LF band, LF/HF is an effective tool to describe the balance between the two parts of the autonomic nervous system. For normal people, if LF/HF is over 2, it means that the person is awake [14]. However, if the ratio of LF and HF is close to 1, it illustrates the person is in non-rapid eye movement (NREM) sleep state [14].

Subsequently, we verified that all the eight BOLD data of subjects were in sleep state by peripheral pulse signal. Thus, it is reasonable to substitute peripheral pulse for EEG, which would make subjects feel more comfortable and easier to fall asleep.

### Altered small-world properties of brain functional networks

Achard et al. [1] indicated that small-world network properties of a large-scale functional brain network with 90 regions were most robust for the 0.03–0.06 Hz frequency band. Accordingly, we analyzed those BOLD images during stable sleep and the results indicated that there existed significant changes in small-world properties of brain network between wake and stable sleep state, and brain network behaved more like small-world pattern during sleep states as compared to the wake state, which was in line with Ferri’s sleep studies using EEG synchronization [9,10]. Actually, small-world featured network makes neural activities more synchronizing in different brain regions and increasing information communication of brain areas, which are also beneficial for brain memory [36–38].

Moreover, results indicated that the topology of brain network altered when humans sleep. From local perspective, higher clustering coefficients meant brain networks held higher local functional interconnections, and thus offered more efficient local information interactions, which were in line with the result of Ferri et al. [10]. Latora and Marchiori reported that local efficiency was mainly associated with short distance connections in adjacent domains which mediated modularized information processing [36]. Therefore, it is reasonable to speculate that under the circumstance that, when subjects are sleep, the paralimbic-limbic and centrencephalic cortex of them are still elastic and robust in local message encoding.

However, in global scope, longer characteristic path length means that information processing in different brain regions becomes slower and less efficient [24,36]. Our results demonstrate that functional brain network with high “cliqueness” in stable sleep is optimal for message encoding, which is consistent with a memory reprocessing hypothesis of Diekelmann and Born [39]. This also can explain why sleep insufficiency and sleep deprivation make adverse impact on memory. It is worth noting that, without EEG monitoring, the interference of BOLD signal and sleep disturbance of subjects can be cut down.

### Changed paralimbic-limbic cortex connectivity from wake to stable sleep state

More interestingly, we observed that paralimbic-limbic cortex had less connectivity with neocortical system and centrencephalic structure in wake state than stable sleep. This finding provides an alternative insights to the prior an H_2_^15^0 PET study on regional cerebral blood flow (rCBF) during deep NREM sleep [14], which reported that activity in paralimbic structures had fallen sharply, i.e. rCBF rates in the anterior insula, temporal polar and anterior cingulate cortices were at their nadir during this period.

We believe that, when humans sleep, the paralimbic-limbic cortex pattern may own an adaptive defense mechanism responsible for suppressing the external environment interference, which is in accord with Braun et al.’s [14] suggestion that the paralimbic-limbic cortex may be functionally disconnected from brain regions which directly mediate their interactions with the external environment. Besides, all findings mentioned above could also explain why sleep is homeostasis which is far less susceptible to outside world.

## Conclusions

In summary, this is the first study, to our knowledge, has observed that small-world properties of brain functional networks altered when human sleeps without EEG monitoring. Moreover, our result showed that paralimbic-limbic cortex was getting more independent during stable sleep state, indicating that graph theory and connectivity graph offer new sight for studying brain’s neural activity when humans sleep.

## Supporting Information

S1 Dataset Raw data. (ZIP)
